# Early Diagnosis of an Atypical Type A Aortic Dissection With Point-of-Care Ultrasound: A Case Report

**DOI:** 10.7759/cureus.67780

**Published:** 2024-08-26

**Authors:** Bastian Rodrigues de Castro, Ivan Peev, Mathilde Dekeuleneer, Florence Dupriez

**Affiliations:** 1 Emergency Department, Hopital Universitaire Saint-Luc Bruxelles, Bruxelles, BEL

**Keywords:** emergency medicine, pocus, point-of-care ultrasound, aortic dissection detection risk score (add-rs), type a aortic dissection, aortic dissection

## Abstract

Aortic dissection is a rare but potentially fatal condition, characterized by a high mortality rate where every minute of delay in treatment counts. Its diagnosis remains challenging due to its often atypical clinical presentation. This case report presents an atypical case of type A aortic dissection in a 75-year-old female patient, highlighting the importance of early diagnosis facilitated by point-of-care ultrasound and emphasizing the value of its use in suspected aortic dissection regardless of the clinical probability. Additionally, this report reviews the risk factors for misdiagnosis and underscores the utility of diagnostic scores such as the aortic dissection detection risk score.

## Introduction

Despite the low annual incidence of 4.8 cases per 100,000 patients [1], aortic dissection must be considered in the differential diagnosis in a wide range of emergency situations due to the high mortality rate associated with misdiagnosis.

Its clinical presentation is highly variable, ranging from typical chest pain to the complete absence of symptoms. Risk factors such as hypertension, smoking, cardiovascular surgery, trauma, or connective tissue diseases may be absent. Clinical examination is frequently non-contributory, hypertension is present in half of cases, and a blood pressure differential between the upper limbs is observed in one-third of cases [2]. Aortic dissection remains a challenging diagnosis to establish in the emergency department (ED).

In a retrospective review, Pare et al. recommend the systematic use of focused cardiac ultrasound in emergency settings to significantly reduce diagnostic delays and minimize misdiagnoses of ascending aortic dissection [3]. This case report presents an atypical case of type A aortic dissection (AAD), highlighting the importance of early diagnosis facilitated by the use of point-of-care ultrasound (PoCUS).

## Case presentation

**Figure 1:**
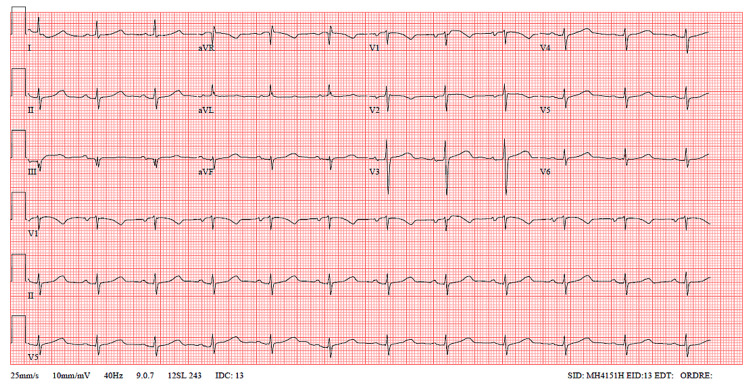
ECG showing sinus rhythm with left anterior hemiblock and QRS fragmentation in lead III and no tachycardia and no ST abnormalities ECG: electrocardiogram

A 75-year-old female patient with no significant medical or surgical history presented to the ED on her own with epigastric abdominal pain that had been present for five hours prior to admission. The pain, described as constrictive and non-radiating, began suddenly when she was getting into her car. The patient reported no tearing sensation, did not take analgesics, and had no chest pain, dyspnea, palpitations, malaise, or loss of consciousness. After her admission to the ED, the pain spontaneously disappeared, leaving only epigastric discomfort. The patient was placed under monitoring. An electrocardiogram revealed a regular sinus rhythm, a left anterior hemiblock with QRS fragmentation in lead III, and no signs of repolarization abnormalities (Figure 1).

**Figure 2:**
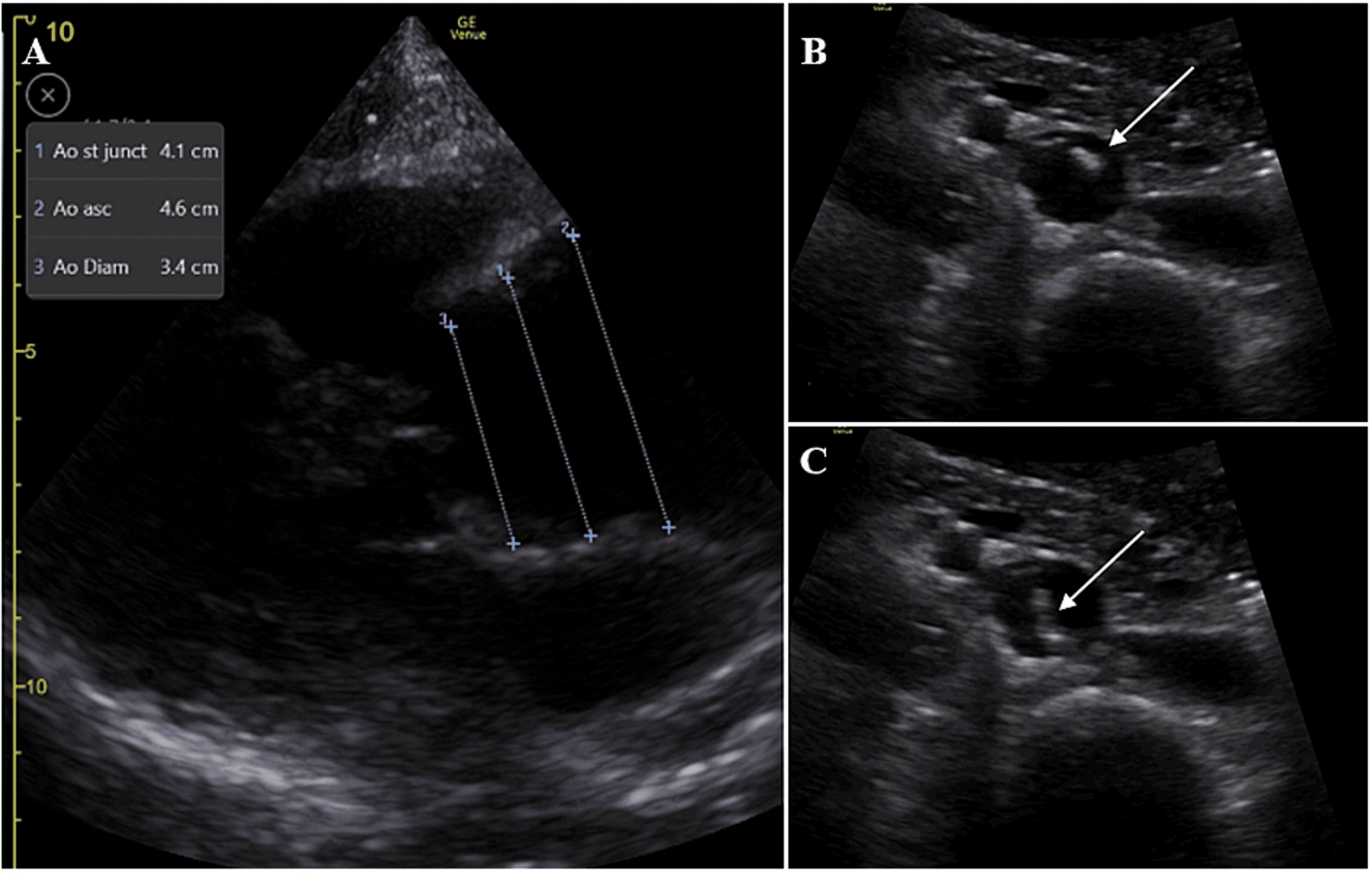
PoCUS showing a dilated aortic root in the parasternal long-axis view (A) and a mobile flap (white arrow) in the abdominal aorta in the transverse view (B, C) PoCUS: point-of-care ultrasound

Vital signs on admission were normal, with no significant difference in blood pressure between arms. The clinical examination, particularly the cardiopulmonary examination, was unremarkable. PoCUS was immediately performed at the patient’s bedside, revealing a 4.5 cm dilation of the aortic root (with a normal range of 2.9 to 3.7 cm in men and 2.7 to 3.5 cm in women) without aortic insufficiency. There was no pericardial effusion, and the left and right ventricular functions were normal. An intimal flap was observed in the abdominal aorta (Figure 2).

**Figure 3:**
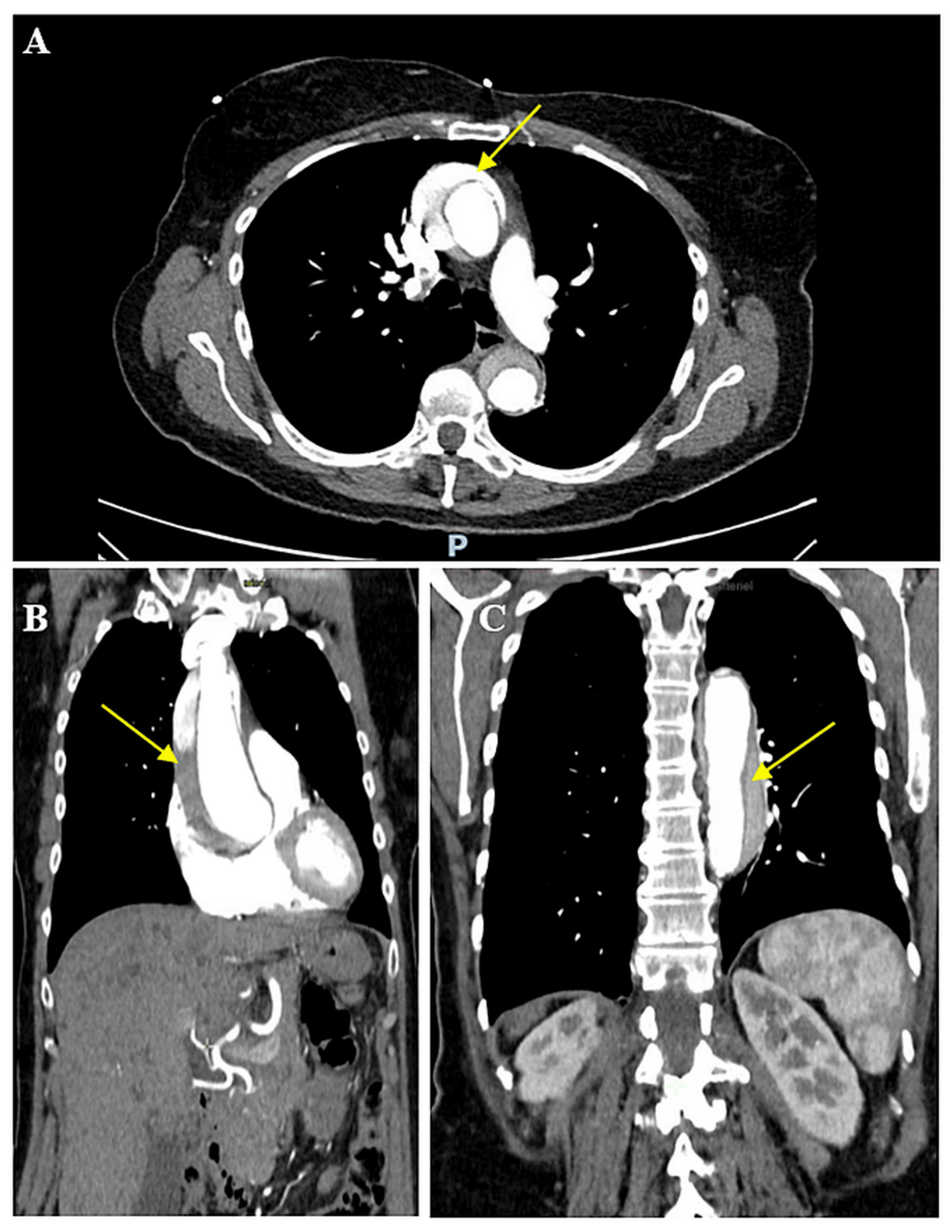
CT angiogram with the axial (A) and coronal (B, C) views showing a Stanford type A thoracoabdominal aortic dissection originating from the focal ulceration of the aortic root, with a spontaneously mural hyperdense hematoma extending 6 cm in an anteroposterior direction. The intimal flap (yellow arrow) extends from the thoracoabdominal aorta to the iliac bifurcation and right external iliac artery CT: computed tomography

Thoracic and vascular surgeons, as well as anesthesiologists, were rapidly contacted due to the strong suspicion of type A aortic dissection. A CT angiogram confirmed a Stanford type A thoracoabdominal aortic dissection, starting with focal ulceration of the aortic root and a mural hyperdense hematoma extending 6 cm in an anteroposterior direction. The intimal flap extended from the thoracoabdominal aorta to the iliac bifurcation and right external iliac artery (Figure 3).

Laboratory results obtained after the CT angiogram revealed no significant abnormalities, with the exception of a significantly elevated D-dimer level of 25,022 ng/mL (Table 1).

**Table 1:** Laboratory findings Significantly elevated D-dimer level GFR: glomerular filtration rate, GOT: glutamic-oxalacetic transaminase, GPT: glutamate-pyruvate transaminase, GGT: gamma-glutamyl transferase

Investigation	Result	Reference Range
Hemoglobin	14.2 g/dL	12.0-15.5 g/dL
White cell count	10,780/µL	4,000-11,000/µL
Platelets	177x10³/mm³	150-410x10³/mm³
Estimated GFR	80 mL/min/1.73m²	>60 mL/min/1.73m²
GOT	24 U/L	7-40 U/L
GPT	12 U/L	7-56 U/L
GGT	30 U/L	9-48 U/L
Total bilirubin	0.5 mg/dL	0.1-1.2 mg/dL
D-dimers	25,022 ng/mL	<500 ng/mL

An emergency surgical intervention was performed, including the partial replacement of the aortic root, ascending aorta replacement, and hemiarch replacement using a 30 mm Vascutek Gelweave Straight Tube prosthesis. The patient was able to return home a few days after the procedure.

## Discussion

The Stanford classification system divides thoracic aortic dissections into two principal types: type A, involving the ascending aorta, and type B, affecting the descending aorta distal to the origin of the left subclavian artery. Stanford type A dissections carry a significantly higher mortality rate compared to type B [4]. Despite the critical urgency of the condition, its diagnosis remains challenging for clinicians due to its often-atypical clinical presentation. Pain may be absent, and clinical examination is frequently non-contributory.

According to the literature review by Lovat et al., misdiagnosis was reported in 33.8% of the 1,663 cases of aortic dissection [5]. Factors contributing to misdiagnosis include patients presenting to the ED on their own [6], clinical presentation compatible with coronary ischemia, heart failure, pulmonary embolism, or stroke, and the absence of mediastinal widening on chest radiography [5-7].

Overall, history taking, clinical examination, and radiography are insufficient to exclude aortic dissection [8]. Factors associated with a more accurate diagnosis include a detailed medical history, D-dimer testing, and increased use of imaging. In a retrospective study published by Ohle et al. in 2019 involving 194 cases of aortic dissection, a comprehensive history detailing the nature of the pain, its severity, duration, and radiation was obtained in only 15% of cases [9].

Clinicians have validated scores such as the aortic dissection detection risk score (ADD-RS) to assist in the diagnostic process. The ADD-RS combines historical and clinical data with D-dimer testing. An ADD-RS score of 0 or ≤1 combined with D-dimers <500 ng/mL allows the exclusion of aortic dissection with sufficient certainty [10-12]. However, D-dimer testing has limitations, including the waiting time for results, its lack of specificity, and its inapplicability for scores >1.

To address these limitations, PoCUS plays a crucial role in managing suspected aortic dissection due to its effectiveness, availability, and rapid use. PoCUS can detect direct and indirect signs of AAD, such as an intimal flap, pericardial effusion, aortic valve regurgitation, aortic dilation >40 mm, or aortic wall thickening [13]. A recent systematic review and meta-analysis by Sutarjono et al., including 9,602 transthoracic echocardiograms in cases of type A aortic dissection, revealed an overall sensitivity of 62.4% (95% CI 60.8-64.0%) and an overall specificity of 87.5% (95% CI 86.7-88.3%) [14]. Due to its limited sensitivity, PoCUS cannot be used as a standalone exclusion test. However, its good specificity facilitates early diagnosis and significantly reduces diagnostic delays [15].

PoCUS plays a critical role not only in cases with a high clinical probability of acute aortic dissection but also in situations with a low clinical probability. In a sub-analysis of the ADvISED study, Nazerian et al. demonstrated that the combination of an ADD-RS score ≤1 with negative PoCUS had a sensitivity of 93.8% (95% CI 88.6-97.1%) and a failure rate of 1.9% (95% CI 0.9-3.6%) for excluding acute aortic syndrome. When this strategy was combined with a negative D-dimer test, the failure rate dropped to 0% (95% CI 0-1.2%) [16]. In the presence of low clinical probability, PoCUS can quickly identify patients needing advanced imaging and strengthen the diagnostic exclusion strategy when combined with a negative D-dimer test. Therefore, integrating clinical echocardiography into the ADD-RS algorithm could be useful in optimizing the diagnosis and management of patients with suspected aortic dissection.

For all these reasons, it seems reasonable to support PoCUS use in the initial evaluation of patients presenting to the ED with suspected aortic dissection, regardless of the clinical probability. Even though contrast echocardiography and transesophageal echocardiography are more effective than PoCUS, they are also more invasive and require more specialized expertise [17].

## Conclusions

It is essential to consider the differential diagnosis of type A aortic dissection, even in the presence of mild or atypical symptoms. PoCUS should be integrated into the management of patients presenting to the ED with suspected aortic dissection, regardless of the clinical probability. Optimizing diagnostic protocols through a combined approach, including the systematic use of PoCUS and the application of clinical scores such as the ADD-RS and D-dimer testing, could help reduce diagnostic errors while minimizing delays in treatment, thereby improving clinical outcomes for patients with suspected aortic dissection. Moreover, further prospective evaluations of this tool are encouraged to facilitate its incorporation into international guidelines for the diagnosis of aortic dissection.
